# QuickStats

**Published:** 2013-02-15

**Authors:** Jiaquan Xu

**Figure:**
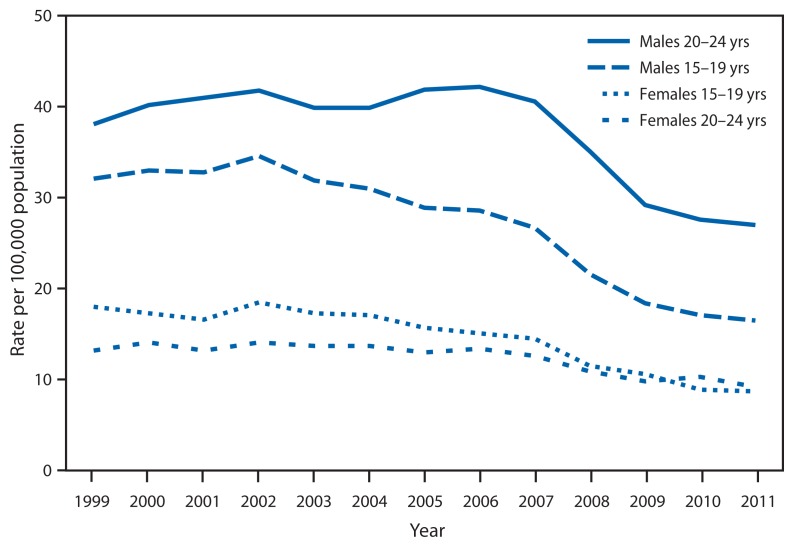
Motor Vehicle Traffic Death Rates*^†^ Among Persons Aged 15–24 Years, by Sex and Age Group — United States, 1999–2011^§^ * Motor vehicle traffic deaths as underlying cause of death are coded to V02–V04 (.1, .9), V09.2, V12–V14 (.3–.9), V19 (.4–.6), V20–V28 (.3–.9), V29–V79 (.4–.9),V80 (.3–.5), V81.1, V82.1, V83–V86 (.0–.3), V87 (.0–.8), and V89.2, according to the *International Classification of Diseases, 10th Revision*. ^†^ Per 100,000 population. The populations used for computing death rates were enumerated as of April 1 for 2000 and 2010, postcensal estimates as of July 1 for 2011, and intercensal estimates as of July 1 for all other years. ^§^ Data for 2011 are preliminary.

From 1999 to 2011, motor vehicle traffic death rates declined by 49% for males aged 15–19 years, 52% for females aged 15–19 years, 29% for males aged 20–24 years, and 30% for females aged 20–24 years. During 1999–2011, the highest rates occurred among males aged 20–24 years, followed by males aged 15–19 years, females aged 15–19 years, and females aged 20–24 years. However, in 2010, the rate for females aged 20–24 years surpassed the rate for females aged 15–19 years.

**Source:** National Vital Statistics System. Mortality public use data files, 1999–2010. Available at http://www.cdc.gov/nchs/data_access/vitalstatsonline.htm. Unpublished mortality data, 2011.

